# Supplementary material for: The cell-wide web coordinates cellular processes by directing site-specific Ca2+ flux across cytoplasmic nanocourses
Source: Nat Commun. 2019 May 24;10:2299. doi: 10.1038/s41467-019-10055-w (PMC6534574; doi:10.1038/s41467-019-10055-w)
Supplement: Supplementary file 1 — Supplementary Information [file 41467_2019_10055_MOESM1_ESM.pdf]

Supplementary information

The cell-wide web coordinates cellular processes by directing site-specific Ca^{2+} flux across cytoplasmic nanocourses

Duan et al.

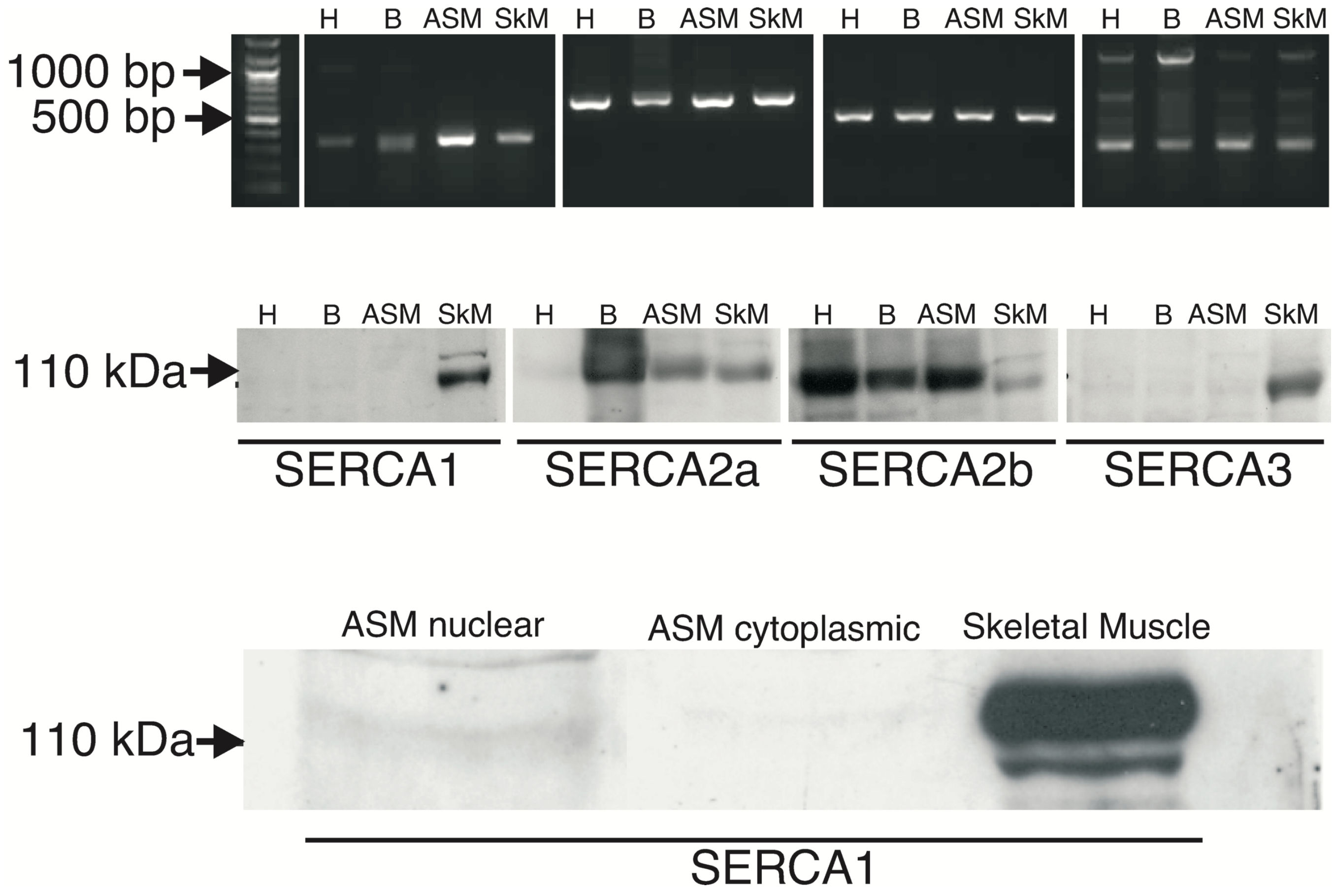

Supplementary Fig. 1 SERCA1 expression and targeting in rat pulmonary artery smooth muscle. Upper panel, RT-PCR fragments of SERCA1, SERCA2a, SERCA2b and SERCA3 amplified from total mRNA extracted from: heart (H), pulmonary arterial smooth muscle (ASM), brain (B) and skeletal muscle (SkM) mRNAs (confirmed in triplicate, n = 3 rats). The predicted sizes of the PCR products were 340 bp for SERCA1, 694 bp for SERCA2a, 501 bp for SERCA2b and 264 bp for SERCA3. Middle panel, Western blots (confirmed in 3 separate experiments) for SERCA1, SERCA2a, SERCA2b and SERCA3 in mircrosomal fractions of lysates from: pulmonary arterial smooth muscle (ASM), brain (B) and skeletal muscle (SkM). Lower panel, Western blots (confirmed in 3 separate experiments, pulmonary arteries from n = 10 rats per replicate, all other tissue n = 1 rat per replicate) for SERCA1 in nuclear fractions of lysates from pulmonary arterial smooth muscle (ASM). Each blot was also probed with an antibody selective for actin to ensure equal protein loading (not shown).

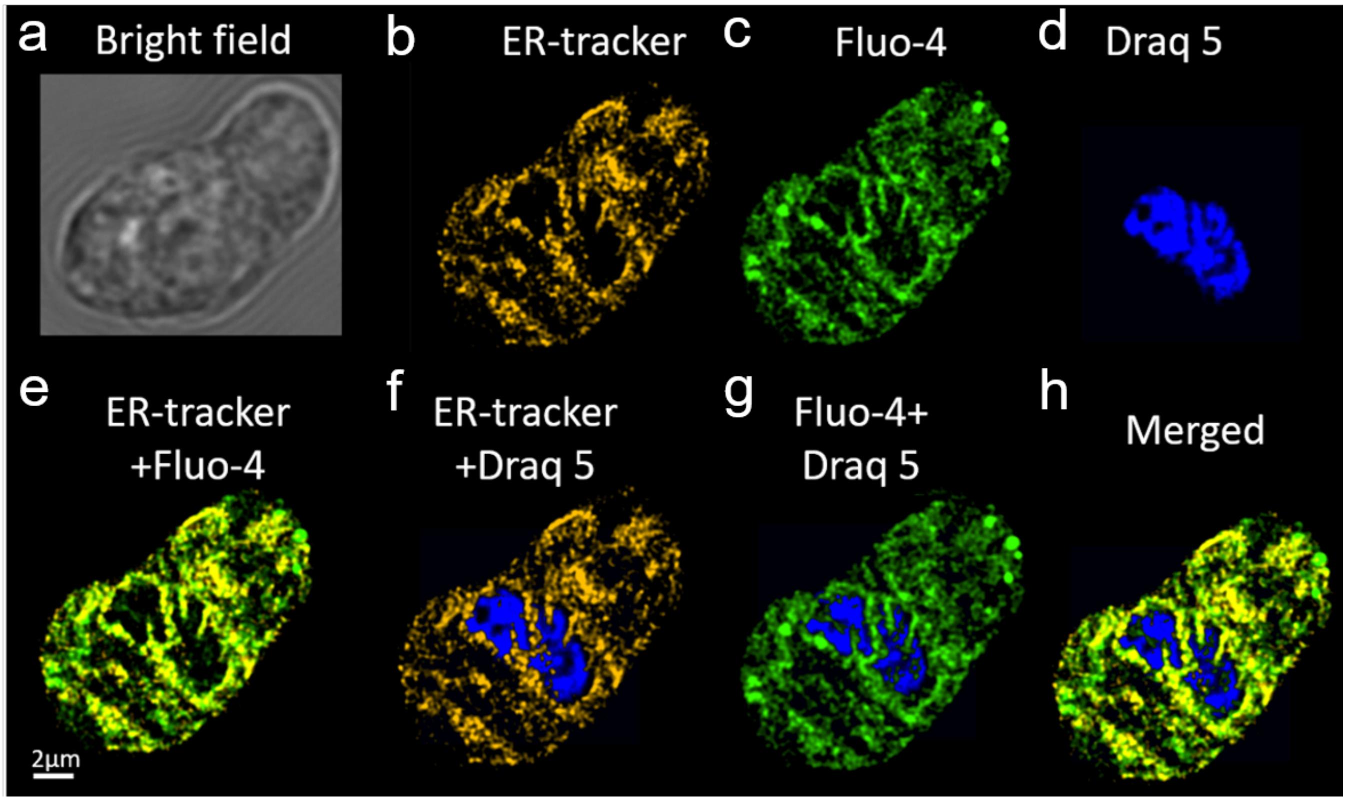

Supplementary Fig. 2 The SR demarcates the cell-wide circuit of cytoplasmic nanocourses from the plasma membrane to the nucleus of acutely isolated pulmonary arterial myocytes. a, Bright field image of an arterial myocyte. b, Deconvolved confocal z section through the middle of the same cell showing ER-Tracker labelling of SR and outer nuclear membrane (Orange). c, As for (b) but showing the distribution of cytoplasmic calcium (Green) as indicated by Fluo-4. d, as for (a) but showing the nucleoplasm identified by Draq5 (blue). e, Merged image of ER-tracker and Fluo-4 labelling. f, Merged image of ER-Tracker and Draq5 labelling. g, Merged image of Fluo-4 and Draq5 labelling. h, Merged image of ER-Tracker, Fluo-4 and Draq5 labelling. Confirmed in n = 8 cells from 3 rats.

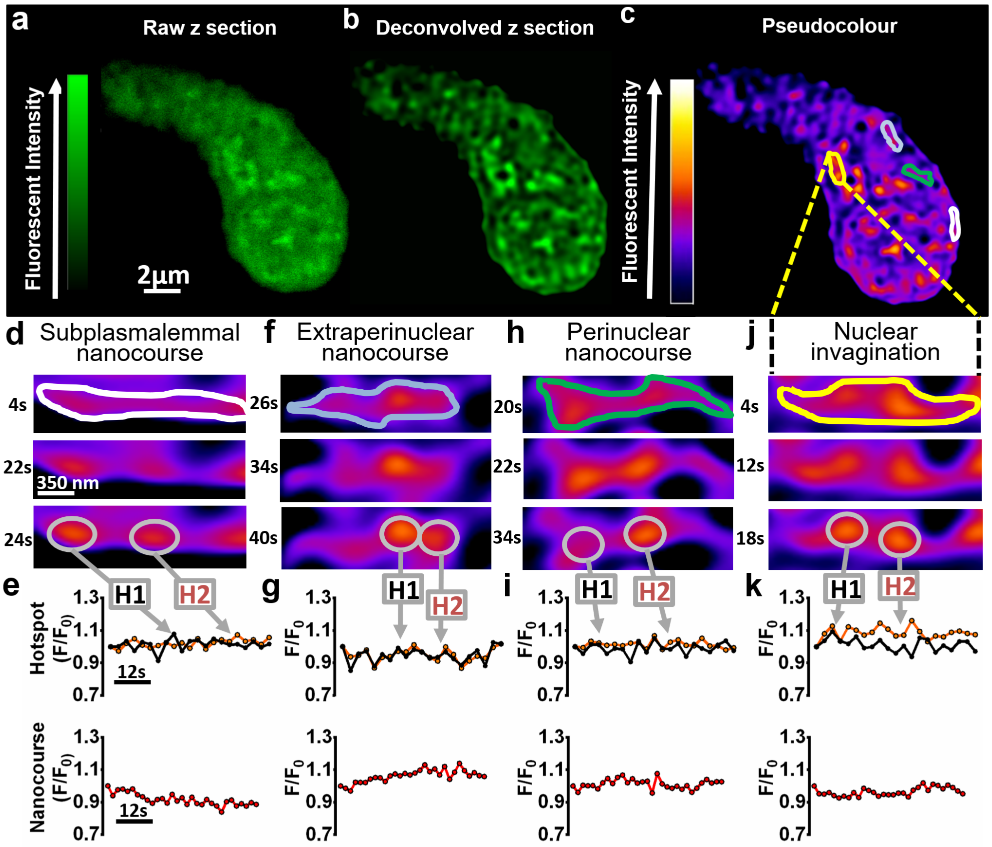

Supplementary Fig. 3 Cytoplasmic nanocourses of acutely isolated arterial myocytes receive Ca^{2+} flux from the SR. a**,** Confocal z section through the centre of a pulmonary arterial smooth muscle cell loaded with Fluo-4 (green) and pre-incubated with 1μM thapsigargin for 1 hour. b, As for (a), but deconvolved. c, as for (b), but pseudocolour applied, indicating relative Fluo-4 fluorescent intensity, with regions of interest identifying exemplar subplasmalemmal (white), extraperinuclear (blue), perinuclear (green) and nuclear nanocourses (yellow). d**,** subplasmalemmal nanocourse identified in (c) is shown at higher magnification and at three different time points (from top to bottom). Gray circles identify two individual "Hotspots" (H1, black; H2, orange) at which Fluo-4 fluorescence varied with time in control cells (see Fig. 1). e, Fluo-4 fluorescence ratio (F_x/F_0) versus time (sampling frequency = 0.5Hz) for H1 and H2 (upper panel) is compared to the average for the whole nanocourse (lower panel). Then, from left to right, extraperinuclear (f and g), perinuclear (h and i), and nuclear (j and k) nanocourses as in (d and e). Confirmed in n = 9 cells from 8 rats. See Fig. 2, main manuscript for average data. Green and pseudocolour look up tables in a and c show relative fluorescence (arbitrary units).

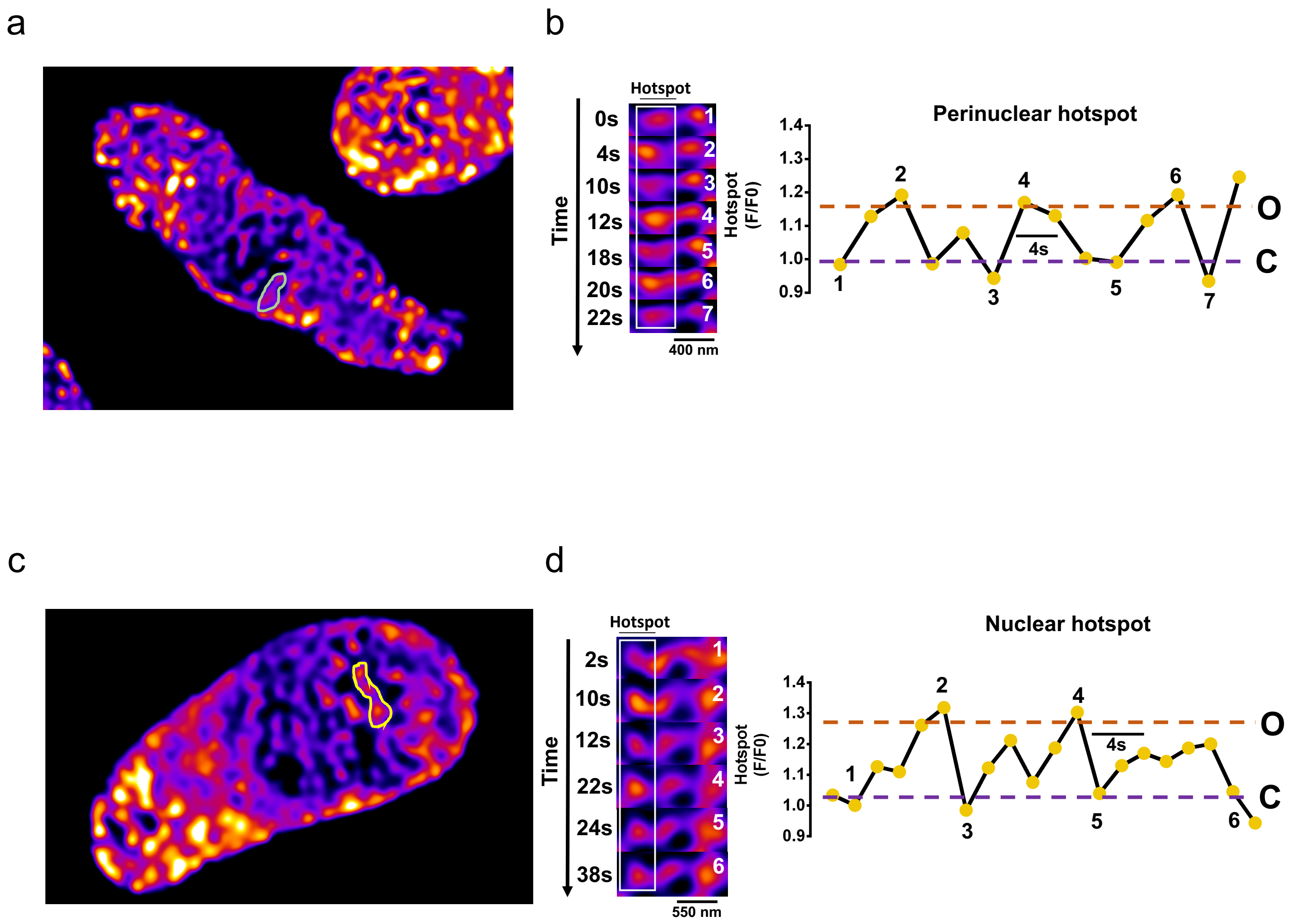

Supplementary Fig. 4 Fluctuating fluorescence intensity for hotspots within cytoplasmic nanocourses. a, Deconvolved confocal z section through the centre of a pulmonary arterial smooth muscle cell loaded with Fluo-4 (pseudocolor applied indicating relative Fluo-4 fluorescent intensity, black = 0, white = maximum). b, Left hand panel shows image time series for the perinuclear nanocourse identified by the blue region of interest at time = 0 shown in (a), right hand panel shows Fluo-4 fluorescence ratio of the hotspot (F_H) divided by F of the nanocourse (F_{N0}) versus time. c and d as in (a and b), but for a different cell and hotspot within a nuclear nanocourse. Confirmed in n = 7 cells from 7 rats. See also Fig. 2, main manuscript.

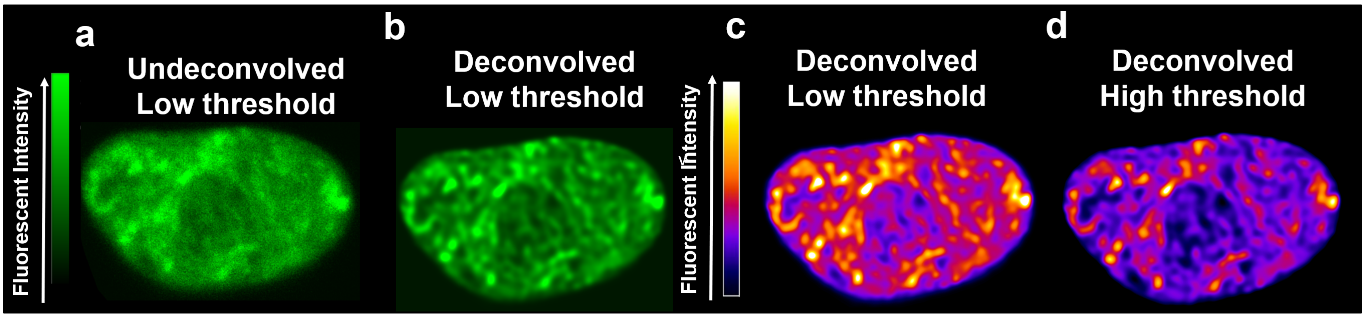

Supplementary Fig. 5 Deconvolution and threshold setting for images prior to Maurocalcine response. a**,** Raw Fluo-4 fluorescence image. b, Deconvolved image with low threshold and low maximum fluorescence (F_{max}) setting, highlighting nanocourse networks. c, Pseudocolour representation of image shown in (b). d, High threshold set with the nucleoplasm F = 0, and a higher F_{max} than in (b) in order to limit signal saturation during cell response to Maurocalcine (see Fig. 3, main manuscript). Green and pseudocolour look up tables in a and c show relative fluorescence (arbitrary units).

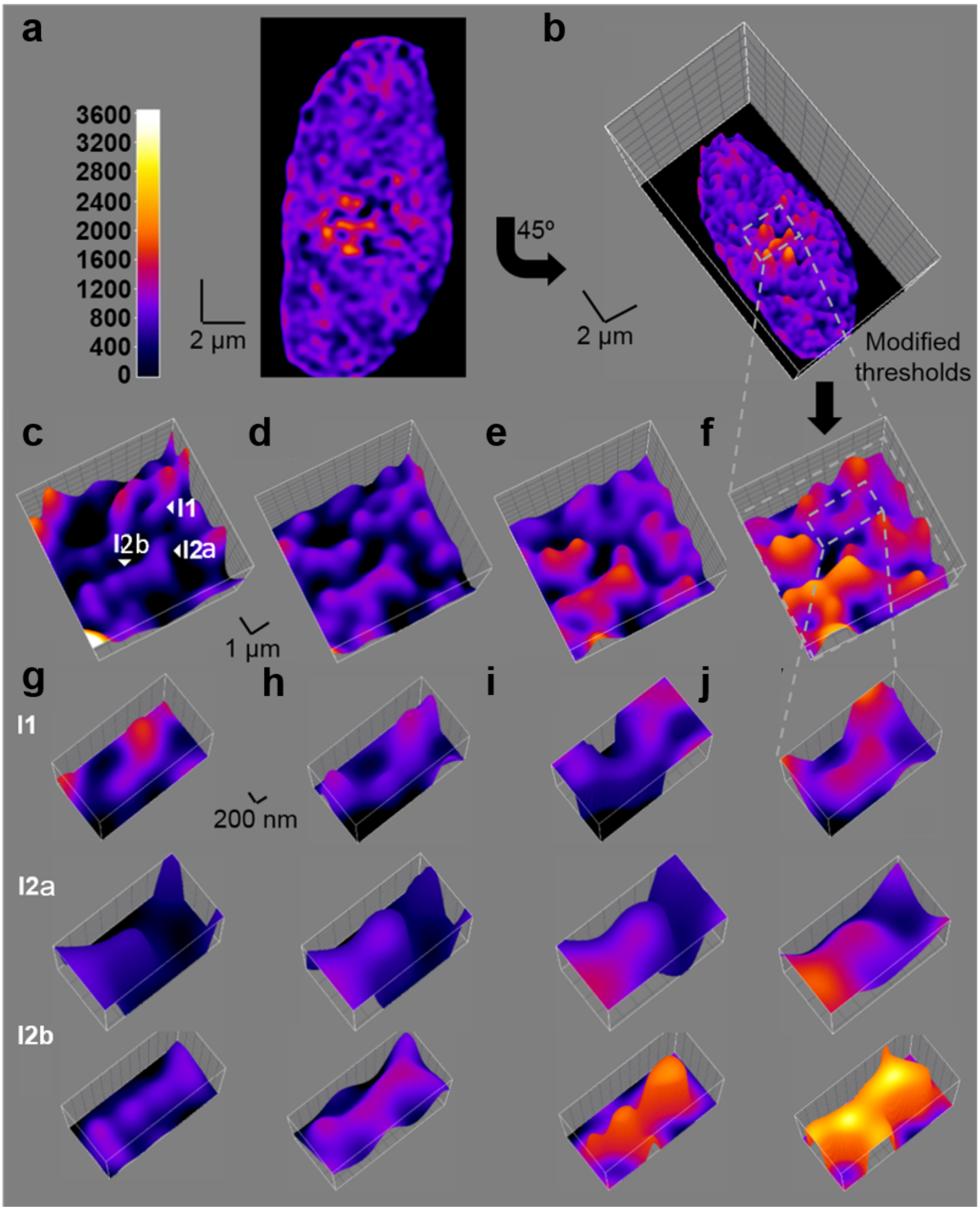

Supplementary Fig. 6 Maurocalcine gates Ca^{2+} flux into nuclear nanocourses. a, Pseudocolour representation of a deconvolved confocal z section through the centre of an arterial myocyte loaded with Fluo-4. b, 3D intensity map of same cell as in (a), 45° rotation. c-f, Higher magnification confocal z section of nuclear region of (b) at (from left to right) different time points before (c) and after (d-f) extracellular application of Maurocalcine (300 nM). g-j, as for (c-f) but showing higher magnification images of each of 3 sites on 2 nuclear nanocourses indicated in (c). Pseudocolour look up table in (a) shows relative fluorescence (arbitrary units).

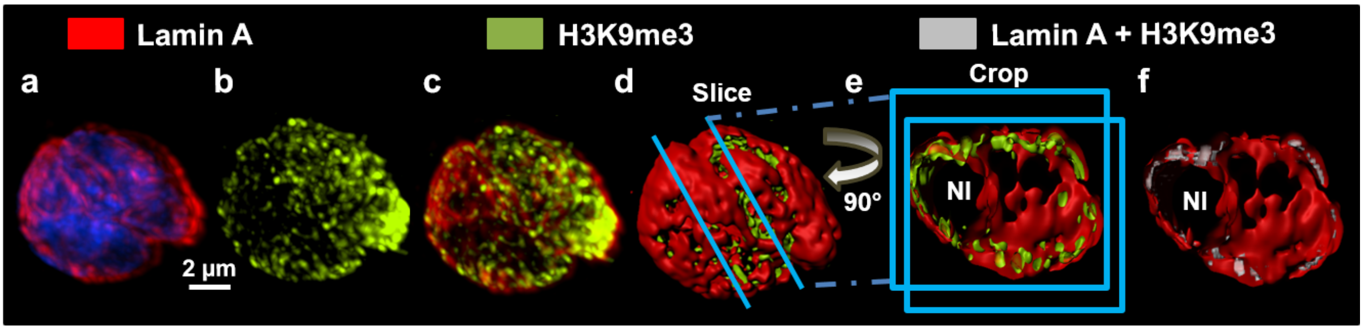

Supplementary Fig. 7 H3K9me3 locates at the outer edge of the nucleus but not in nuclear invaginations. a, 3D reconstruction of a deconvolved z stack of confocal images through the nucleus of a pulmonary arterial myocyte labelled for lamin A. The nucleoplasm is identified by DAPI (blue). b, As for (a), but showing labelling for H3K9me3 (olive) without DAPI. c, Merged image showing lamin A (red) and H3K9me3 (olive) labelling. d, As for (c), but with a digital skin. e, Transverse section through the middle of the nucleus in d, rotated to show nuclear invaginations (NI). f, as in e, but showing lamin A (red) labelled regions colocalised with H3K9me3 in light grey. Confirmed in n = 6 cells from 3 rats.

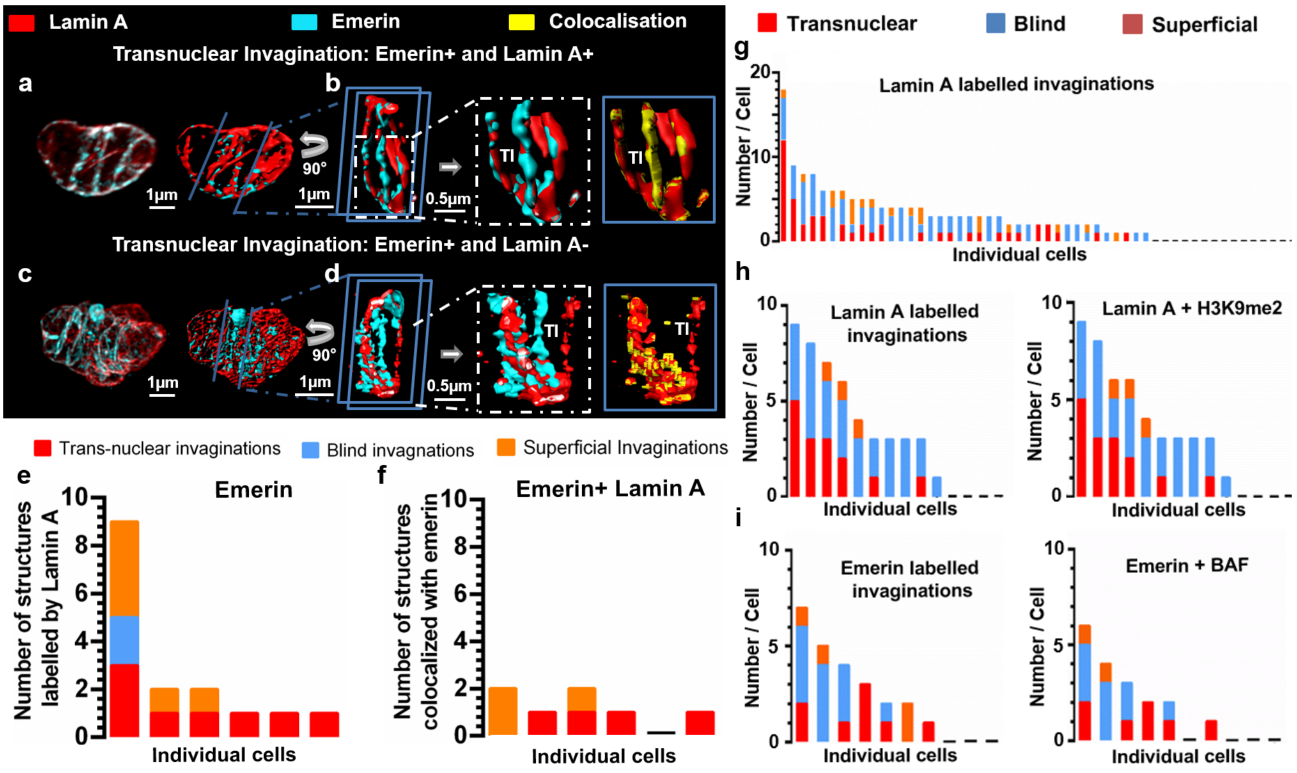

Supplementary Fig. 8 Emerin lines lamin A positive and negative nuclear invaginations. a, 3D reconstruction of a deconvolved z stack of confocal images through the nucleus of an arterial myocyte labelled for lamin A (red) and emerin (cyan) with (right) and without (left) the application of a digital skin. b, Transverse section through the 3D reconstruction in (a) rotated (left) to identify a transnuclear invagination (TI), and a further section at higher magnification (middle), with regions of co-localisation shown in yellow (right). c and d, As in (a and b), but showing an emerin positive and lamin A negative nuclear invagination. e, Stack bar chart shows the number of emerin positive invaginations for each cell. f, As for (e), but for nuclear invaginations positive for both lamin A and emerin (n = 6 from 3 rats). g, stack bar chart shows number of lamin A labelled nuclear invaginations by type, per cell (n = 54 cells from 14 rats) in acutely isolated cells. h, stack bar charts show number of (left hand panel) lamin A labelled nuclear invaginations and lamin A and H3K9me2 positive nuclear invaginations by type, per cell (n = 14 cells from 3 rats) in a different set of acutely isolated cells. i as for (f), but for emerin and BAF (n = 10 cells from 3 rats).

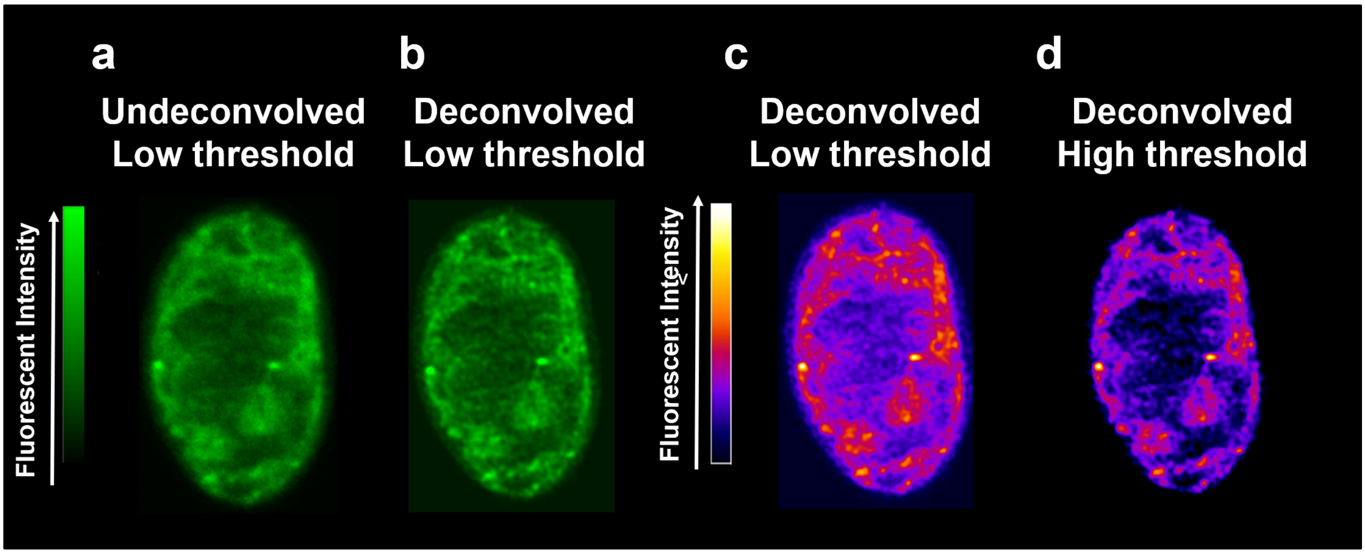

Supplementary Fig. 9 Deconvolution and threshold setting of images prior to Angiotensin II response. a, Raw Fluo-4 fluorescence image. b, Deconvolved image with low threshold and low maximum fluorescence (F_{max}) setting, highlighting nanocourse networks. c, Pseudocolour representation of image shown in (b). d, High threshold set with the nucleoplasm F = 0, with a higher F_{max} than in (b) in order to limit signal saturation during cell response to Angiotensin II (see Figure 6, main manuscript). Green and pseudocolour look up tables in a and c show relative fluorescence (arbitrary units).

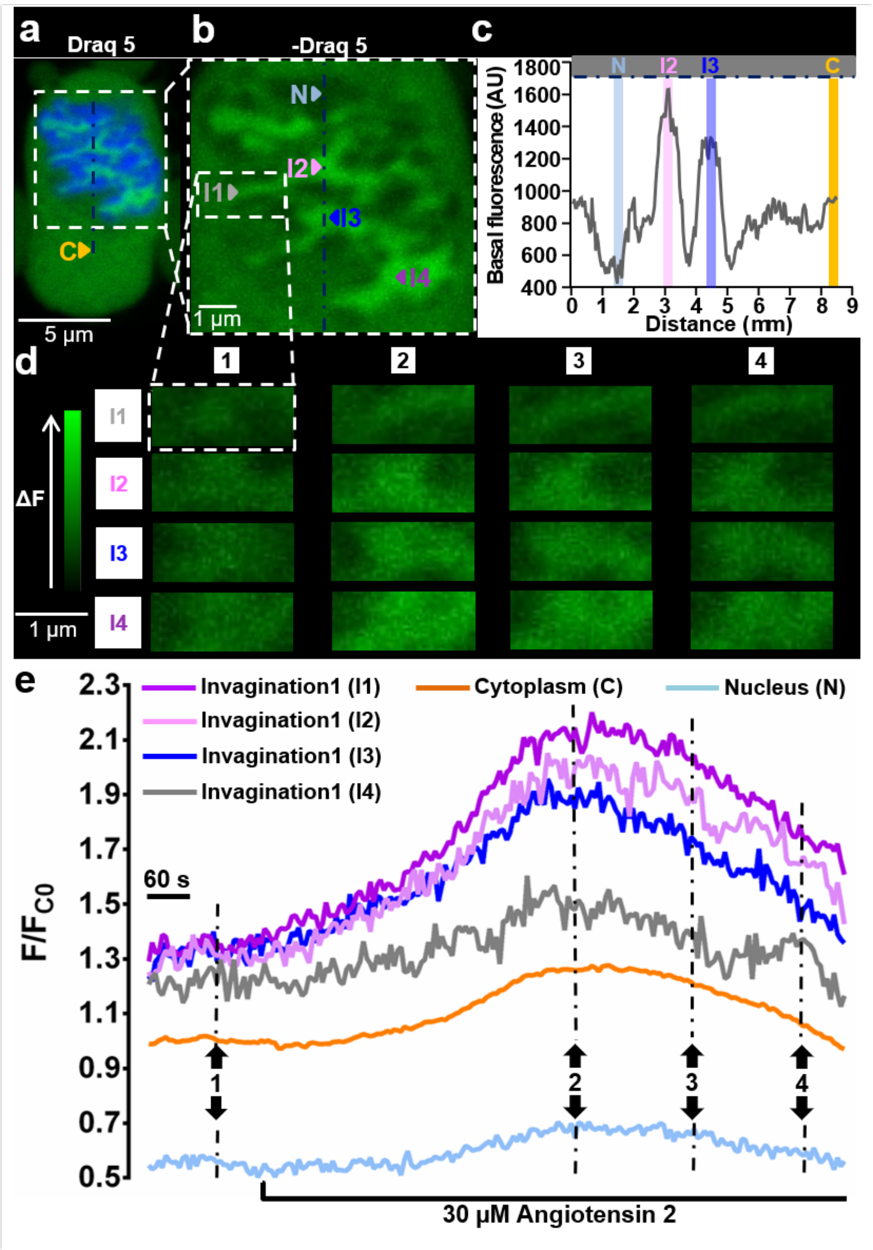

Supplementary Fig. 10 Angiotensin II induces Ca^{2+} signals that exhibit different kinetics in the cytoplasm, nuclear nanocourses and nucleoplasm. a, Raw confocal z section through the centre of an arterial myocyte loaded with Fluo-4 (green). b, enlarged section of same cell to show the nuclear nanocourses. c, 3D intensity map across image in (b, see blue dotted line). d, nuclear invaginations identified in (b) at different time points during application of 30 µM Angiotensin II. e, Time course for Ca^{2+} transient in response to 30 µM Angiotensin II in the cytoplasm (C), nuclear nanocourses (I1-4) and nucleoplasm (N). Green look up table in (d) shows relative fluorescence (arbitrary units). Confirmed in n = 20 cells, from 15 rats.

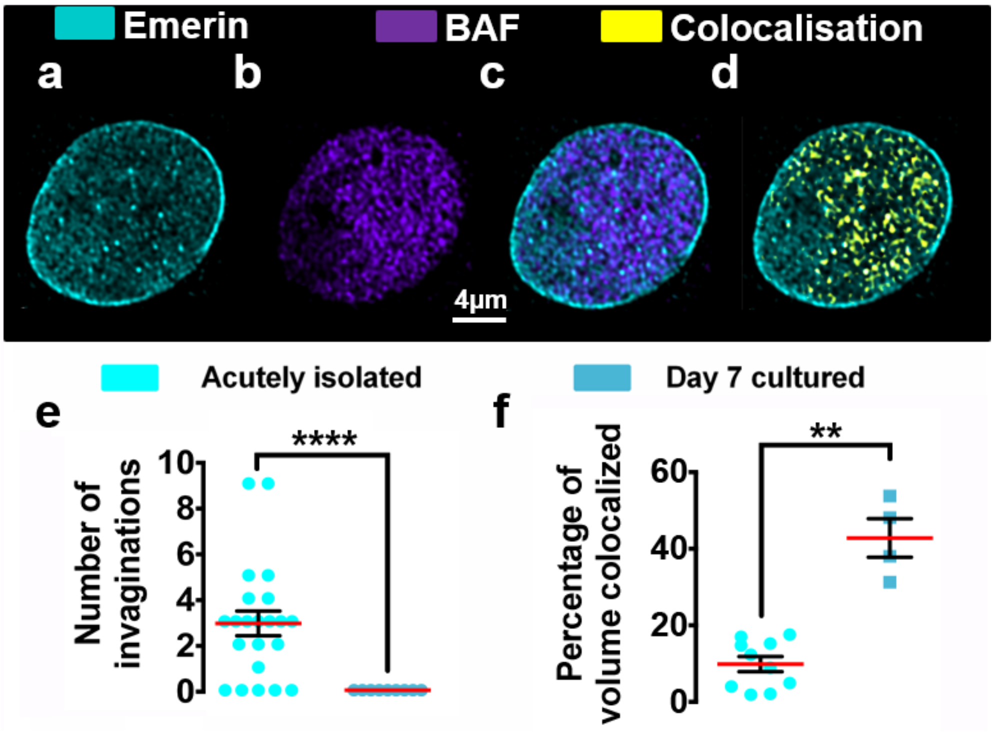

Supplementary Fig. 11 During myocyte proliferation emerin labelled nuclear invaginations are lost and emerin/BAF attachment points reconfigured. a, Deconvolved confocal z section through the middle of the nucleus of a proliferating arterial myocyte labelled for emerin (cyan). b, Same section of cell labelled for barrier-to-autointegration factor (BAF, purple). c, Merged image showing emerin (cyan) and BAF (purple) labelling. d, Merged image showing emerin (cyan) labelling and regions of co-localisation with BAF (yellow). e, Dot plot shows the number of emerin labelled nuclear invaginations per cell for acutely isolated arterial myocytes (cyan; n = 10 cells from 3 rats) and day 7 cultured cells (blue; n = 4 cells from 3 rats). f, Dot plot shows the percentage of volume of emerin labelling colocalised with BAF in acutely isolated arterial myocytes (cyan) and day 7 cultured cells (blue). One-way ANOVA followed by Dunnett's multiple comparisons test: *=p<0.05, **=p<0.01, *** =P<0.001, ****=p< 0.0001.

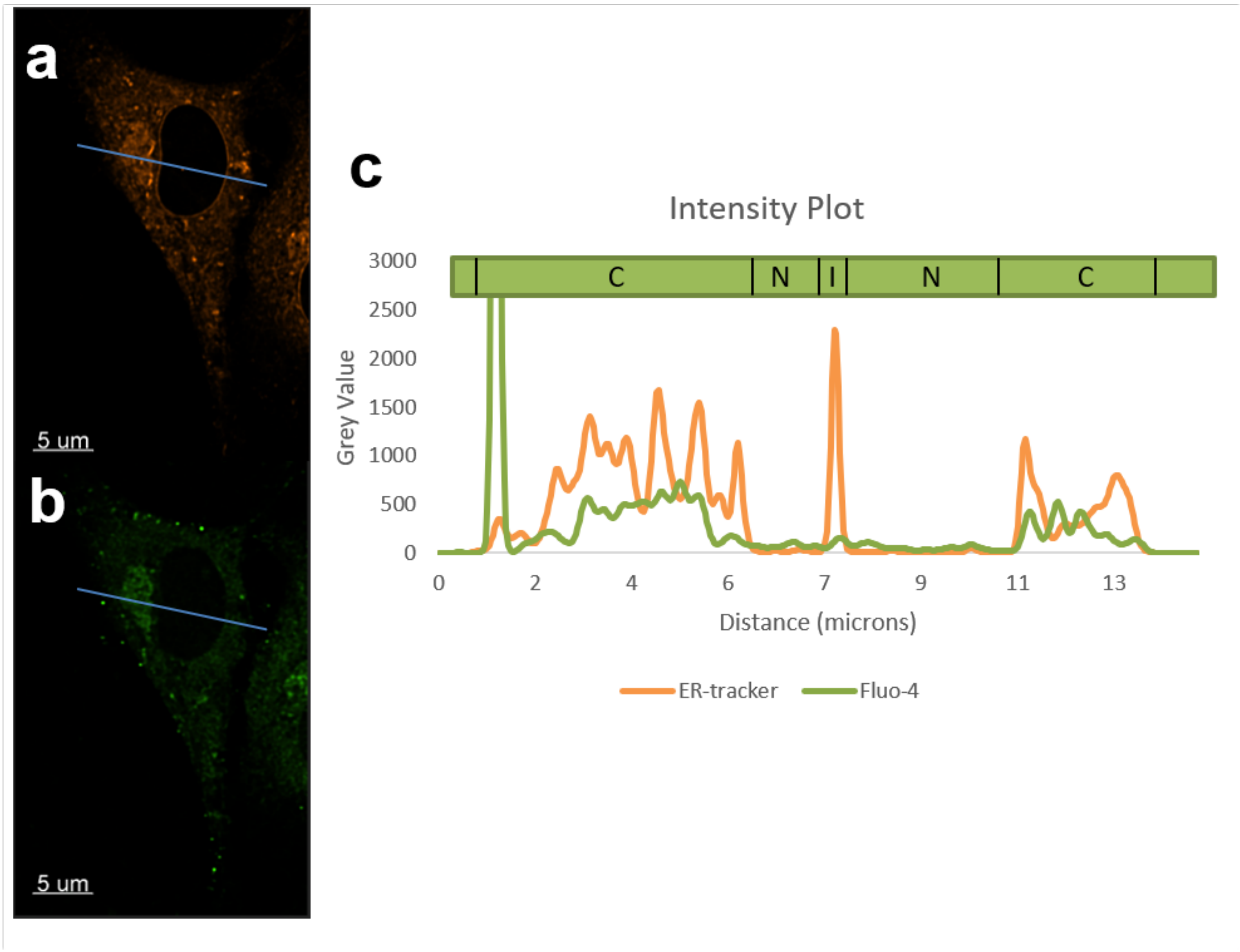

Supplementary Fig. 12 Nuclear invagination identified by ER tracker in cultured cell. a, Deconvolved confocal z section through the centre of a cultured pulmonary arterial myocyte loaded with ER Tracker (orange). b, Deconvolved confocal z section through the centre of the same cell showing Fluo-4 fluorescence (green). c, Intensity plot for ER Tracker (orange) and Fluo-4 (green) taken along the blue line indicated across the cell shown in (a) and (b); C, cytoplasm; N, nucleoplasm; I, nuclear invagination.

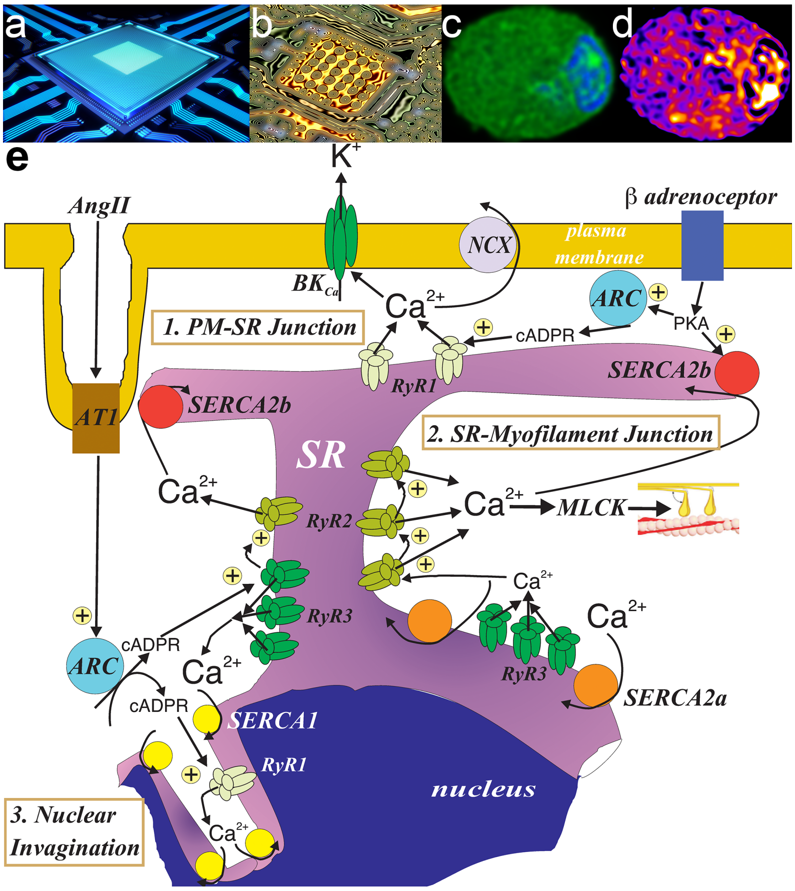

Supplementary Fig. 13 Calcium signalling is analogous to quantum tunnelling across a cell-wide circuit with the nucleus at its centre. a and b, Microprocessor at the centre of a circuit board. c (Fluo-4, green) and d (Fluo-4, pseudocolour), Nucleus (blue) at the centre of a cell-wide circuit of cytoplasmic nanocourses. e, Schematic shows Ca^{2+} flux across cytoplasmic nanocourses demarcated by junctions between the plasma membrane and sarcoplasmic reticulum (PM-SR junction), the sarcoplasmic reticulum aligned with the contractile myofilaments and the nuclear invaginations. Angiotensin II, AngII; Ryanodine receptor, RyR; Sarco/endoplasmic reticulum Ca^{2+} ATPase, SERCA; Na^{2+}/Ca^{2+} exchanger, NCX; ADP-ribosyl cyclase, ARC; Myosin light chain kinase, MLCK; AT1, Angiotensin AT1 receptor; Protein kinase A PKA; large conductance Ca^{2+}-activated potassium channel, BK_{Ca}. Panels (a) and (b) are free images. The schematic in panel (e) was adapted from previous versions developed and published by AME. Reprinted from Publication title, Vol /edition number, Author(s), Title of article / title of chapter, Pages No., Advances in Pharmacology*,* 78, Evans AM, Nanojunctions of the Sarcoplasmic Reticulum Deliver Site- and Function-Specific Calcium Signaling in Vascular Smooth Muscles, 1-47 Copyright (2017), with permission from Elsevier.

Supplementary Movie 1 Hotspots of Ca^{2+} flux within cytoplasmic nanocourses of an acutely isolated arterial myocyte (nucleoplasm Fluorescence set to zero).

Supplementary Movie 2 Hotspots of Ca^{2+} flux within a subplasmalemmal nanocourse of an acutely isolated arterial myocyte.

Supplementary Movie 3 Hotspots of Ca^{2+} flux within a perinuclear nanocourse of an acutely isolated arterial myocyte.

Supplementary Movie 4 Hotspots of Ca^{2+} flux within a nuclear nanocourse of an acutely isolated arterial myocyte.

Supplementary Movie 5 LysoTracker labelled endolysosomes migrate through cytoplasmic naonocourses.

Supplementary Movie 6 Static clusters of mitochondria within cytoplasmic nanocourses, labelled with Mitotacker Red.

Supplementary Movie 7 The RyR1 agonist Maurocalcine selectively promotes Ca^{2+} flux into subplasmalemmal nanocourses and promotes relaxation of a pulmonary arterial myocyte.

Supplementary Movie 8 3D intensity map versus time showing that Maurocalcine induces functionally segregated Ca^{2+} signals within nuclear nanocourses (nucleoplasm Fluorescence set to zero).

Supplementary Movie 9 3D intensity map versus time showing that Angiotensin II induces functionally segregated Ca^{2+} signals within nuclear nanocourses (nucleoplasm Fluorescence set to zero).

Supplementary Movie 10 Supplementary Movie 10. Angiotensin II induced propagating Ca^{2+} wave in extraperinuclear and perinuclear nanocourse and triggers contraction of an arterial myocyte.
