# Supplementary material for: The cell-wide web coordinates cellular processes by directing site-specific Ca2+ flux across cytoplasmic nanocourses
Source: Nat Commun. 2019 May 24;10:2299. doi: 10.1038/s41467-019-10055-w (PMC6534574; doi:10.1038/s41467-019-10055-w)
Supplement: Supplementary file 3 — Reporting Summary [file 41467_2019_10055_MOESM3_ESM.pdf]

nature research

Corresponding author(s): *Double-blind peer review submissions: write DBPR and your manuscript number here instead of author names.*

Last updated by author(s): *YYYY-MM-DD*

Reporting Summary

Nature Research wishes to improve the reproducibility of the work that we publish. This form provides structure for consistency and transparency in reporting. For further information on Nature Research policies, see Authors & Referees and the Editorial Policy Checklist.

Statistics

For all statistical analyses, confirm that the following items are present in the figure legend, table legend, main text, or Methods section.

n/a	Confirmed	
☐	☒	The exact sample size (n) for each experimental group/condition, given as a discrete number and unit of measurement
☐	☒	A statement on whether measurements were taken from distinct samples or whether the same sample was measured repeatedly
☐	☒	The statistical test(s) used AND whether they are one- or two-sided<br>*Only common tests should be described solely by name; describe more complex techniques in the Methods section.*
☒	☐	A description of all covariates tested
☐	☒	A description of any assumptions or corrections, such as tests of normality and adjustment for multiple comparisons
☐	☒	A full description of the statistical parameters including central tendency (e.g. means) or other basic estimates (e.g. regression coefficient) AND variation (e.g. standard deviation) or associated estimates of uncertainty (e.g. confidence intervals)
☐	☒	For null hypothesis testing, the test statistic (e.g. F, t, r) with confidence intervals, effect sizes, degrees of freedom and P value noted<br>*Give P values as exact values whenever suitable.*
☒	☐	For Bayesian analysis, information on the choice of priors and Markov chain Monte Carlo settings
☒	☐	For hierarchical and complex designs, identification of the appropriate level for tests and full reporting of outcomes
☒	☐	Estimates of effect sizes (e.g. Cohen's d, Pearson's r), indicating how they were calculated

Our web collection on statistics for biologists contains articles on many of the points above.

Software and code

Policy information about availability of computer code

Data collection	Image processing was carried out using Rasband WS. ImageJ, U.S. National Institutes of Health, Bethesda, Maryland, USA, imagej.nih.gov/ij/, 1997—2012, Softworx acquisition and analysis software (Applied Precision, UK), Image processing and 3D rendering was carried out using Imaris (Bitplane, Oxford Instruments, UK), Volocity software (Perkin-Elmer, UK), Huygens Essential (Scientific Volume Imaging, Netherland),
Data analysis	Image analysis was carried out using Rasband WS. ImageJ, U.S. National Institutes of Health, Bethesda, Maryland, USA, imagej.nih.gov/ij/, 1997—2012 and Volocity software (Perkin-Elmer, UK), Graphpad Prism, Rasband WS. ImageJ, U.S. National Institutes of Health, Bethesda, Maryland, USA, imagej.nih.gov/ij/, 1997—2012. Data analysis was carried out using Graphpad Prism (Graphpad Software Inc., USA) and MINITAB 14.

For manuscripts utilizing custom algorithms or software that are central to the research but not yet described in published literature, software must be made available to editors/reviewers. We strongly encourage code deposition in a community repository (e.g. GitHub). See the Nature Research guidelines for submitting code & software for further information.

Data

Policy information about availability of data

All manuscripts must include a data availability statement. This statement should provide the following information, where applicable:

- Accession codes, unique identifiers, or web links for publicly available datasets
- A list of figures that have associated raw data
- A description of any restrictions on data availability

The data sets generated during and/or analysed during the current study are available from the corresponding author on reasonable request.

Field-specific reporting

Please select the one below that is the best fit for your research. If you are not sure, read the appropriate sections before making your selection.

☒ Life sciences ☐ Behavioural & social sciences ☐ Ecological, evolutionary & environmental sciences

For a reference copy of the document with all sections, see nature.com/documents/nr-reporting-summary-flat.pdf

Life sciences study design

All studies must disclose on these points even when the disclosure is negative.

Sample size	*Describe how sample size was determined, detailing any statistical methods used to predetermine sample size OR if no sample-size calculation was performed, describe how sample sizes were chosen and provide a rationale for why these sample sizes are sufficient.*
Data exclusions	No data were excluded. However, cells under investigation were excluded if they contracted to such an extent that accurate analysis was compromised. Experiments were also terminated and excluded if cells were compromised / killed by laser-dependent phototoxicity.
Replication	At least 3 repeats were performed on samples form at least 3 different animals
Randomization	rats, tissue and cell suspensions/cultures were selected for experimental work randomly.
Blinding	All data were analysed by the lead experimenter and then checked blinded by the Principle Investigator and one other co-worker.

Reporting for specific materials, systems and methods

We require information from authors about some types of materials, experimental systems and methods used in many studies. Here, indicate whether each material, system or method listed is relevant to your study. If you are not sure if a list item applies to your research, read the appropriate section before selecting a response.

Materials & experimental systems

n/a	Involved in the study	
☐	☒	Antibodies
☒	☐	Eukaryotic cell lines
☒	☐	Palaeontology
☐	☒	Animals and other organisms
☒	☐	Human research participants
☒	☐	Clinical data

Methods

n/a	Involved in the study	
☒	☐	ChIP-seq
☒	☐	Flow cytometry
☒	☐	MRI-based neuroimaging

Antibodies

Antibodies used

Primary antibodies used were as follows:
SERCA antibodies - SERCA1, mouse monoclonal, raised against residues 199–505 of rabbit SERCA1 (Abcam). SERCA2a, rabbit polyclonal, raised against residues 989–997 of pig SERCA2a17, a kind gift from F. Wuytack, University of Leuven, Belgium. SERCA2b, rabbit polyclonal, raised against residues 1032–1043 of pig SERCA2b17, a kind gift from F. Wuytack, University of Leuven, Belgium. SERCA3, rabbit polyclonal, raised against residues 29–39 of mouse SERCA3 (Abcam).
RyR antibodies - RyR1, affinity-purified rabbit anti-RyR1 raised against the peptide residues 4476-448663. RyR2, anti-RyR2 raised against the peptide residues 1344-136564. RyR3, anti-RyR3 raised against the peptide residues 4236-433665. All were a kind gift from S. Fleicher, Vanderbilt University, Tennessee, USA.
Nuclear envelope proteins - Emerin polyclonal antibody were a kind gift from Glenn Morris and outcomes confirmed by use of commercial emerin polyclonal antibodies (Invitrogen, catalogue number: PA5-51424). Thoroughly characterised, see http://www.glennmorris.org.uk/mabs/WCIND.htm).
Lamin A polyclonal antibody (ABCAM; catalogue number: ab26300), specificity previously confirmed66.
Histone H3 di methyl K9 (ABCAM; catalogue number: ab194680). Di-Methyl-Histone H3 Lys9 polyclonal antibody (Invitrogen; catalogue number: PA5-16195). Epigenetic mark antibodies were selected by loss of labelling in yeast with enzymes that confer the marks deleted.
Secondary antibodies used were as follows:
Texas Red-conjugated goat anti-rabbit secondary (Jackson ImmunoResearch, USA; cataologue number: 200-072-211).
Alexa Fluor® 488-AffiniPure goat anti-rabbit IgG (H+L) (Stratech Scientific Limited for Jackson ImmunoResearch; catalogue number: 111-545-144-JIR).
Alexa Fluor® 546 goat anti-mouse IgG (H+L), highly cross-adsorbed (Life Technologies; catalogue number: A-11030).
Alexa Fluor® 488 donkey anti-mouse IgG (H+L) highly cross-adsorbed (Invitrogen; catalogue number: A-21202).
Alexa Fluor 568 donkey anti-rabbit IgG (H+L) highly cross-adsorbed, (Invitrogen; catalogue number: A10042).

Validation

Citations are provided for validation of bespoke antibodies: RyRs = Jeyakumar LH, et al. The skeletal muscle ryanodine receptor

isoform 1 is found at the intercalated discs in human and mouse hearts. J Muscle Res Cell Motil 23, 285-292 (2002).

66. Jeyakumar LH, et al. FKBP binding characteristics of cardiac microsomes from diverse vertebrates. Biochem Biophys Res Commun 281, 979-986 (2001).

67. Jeyakumar LH, et al. Purification and characterization of ryanodine receptor 3 from mammalian tissue. J Biol Chem 273, 16011-16020 (1998); SERCA:

61. Wuytack F, Eggermont JA, Raeymaekers L, Plessers L, Casteels R. Antibodies against the non-muscle isoform of the endoplasmic reticulum Ca2(+)-transport ATPase. Biochem J 264, 765-769 (1989).

62. Eggermont JA, Wuytack F, Verbist J, Casteels R. Expression of endoplasmic-reticulum Ca2(+)-pump isoforms and of phospholamban in pig smooth-muscle tissues. Biochem J 271, 649-653 (1990).

Lamin A polyclonal antibody (ABCAM; catalogue number: ab26300), specificity previously confirmed66.
Nuclear envelope proteins - Emerin polyclonal antibody were a kind gift from Glenn Morris and outcomes confirmed by use of commercial emerin polyclonal antibodies (Invitrogen, catalogue number: PA5-51424). Thoroughly characterised, see http://www.glennmorris.org.uk/mabs/WCIND.htm).

Histone H3 di methyl K9 (ABCAM; catalogue number: ab194680). Di-Methyl-Histone H3 Lys9 polyclonal antibody (Invitrogen; catalogue number: PA5-16195). Epigenetic mark antibodies were selected by loss of labelling in yeast with enzymes that confer the marks deleted.

Validation summaries for commercial antibodies are available from the supplier.

Animals and other organisms

Policy information about studies involving animals; ARRIVE guidelines recommended for reporting animal research

Laboratory animals	Adult male Sprague Dawley rats (~300 g)
Wild animals	NA
Field-collected samples	NA
Ethics oversight	University of Edinburgh ethical approval committee, All experiments were performed under the United Kingdom Animals (Scientific Procedures) Act 1986

Note that full information on the approval of the study protocol must also be provided in the manuscript.
