# Supplementary material for: The cell-wide web coordinates cellular processes by directing site-specific Ca2+ flux across cytoplasmic nanocourses
Source: Nat Commun. 2019 May 24;10:2299. doi: 10.1038/s41467-019-10055-w (PMC6534574; doi:10.1038/s41467-019-10055-w)
Supplement: Supplementary file 4 — Description of Additional Supplementary Files [file 41467_2019_10055_MOESM4_ESM.docx]

**Description of Additional Supplementary Files**

File Name: Supplementary Movie 1

Description: Hotspots of Ca2+ flux within cytoplasmic nanocourses of an acutely isolated arterial myocyte (nucleoplasm Fluorescence set to zero)

File Name: Supplementary Movie 2

Description: Hotspots of Ca2+ flux within a subplasmalemmal nanocourse of an acutely isolated arterial myocyte.

File Name: Supplementary Movie 3

Description: Hotspots of Ca2+ flux within a perinuclear nanocourse of an acutely isolated arterial myocyte.

File Name: Supplementary Movie 4

Description: Hotspots of Ca2+ flux within a nuclear nanocourse of an acutely isolated arterial myocyte.

File Name: Supplementary Movie 5

Description: LysoTracker labelled endolysosomes migrate through cytoplasmic naonocourses.

File Name: Supplementary Movie 6

Description: Static clusters of mitochondria within cytoplasmic nanocourses, labelled with Mitotacker Red.

File Name: Supplementary Movie 7

Description: The RyR1 agonist Maurocalcine selectively promotes Ca2+ flux into subplasmalemmal nanocourses and promotes relaxation of a pulmonary arterial myocyte.

File Name: Supplementary Movie 8

Description: 3D intensity map versus time showing that Maurocalcine induces functionally segregated Ca2+ signals within nuclear nanocourses (nucleoplasm Fluorescence set to zero).

File Name: Supplementary Movie 9

Description: 3D intensity map versus time showing that Angiotensin II induces functionally segregated Ca2+ signals within nuclear nanocourses (nucleoplasm Fluorescence set to zero).

File Name: Supplementary Movie 10

Description: Angiotensin II induced propagating Ca2+ wave in extraperinuclear and perinuclear nanocourse and triggers contraction of an arterial myocyte.
